# Continuous increase of vancomycin resistance in enterococci causing nosocomial infections in Germany − 10 years of surveillance

**DOI:** 10.1186/s13756-018-0353-x

**Published:** 2018-04-24

**Authors:** Cornelius Remschmidt, Christin Schröder, Michael Behnke, Petra Gastmeier, Christine Geffers, Tobias Siegfried Kramer

**Affiliations:** 1 Institute of Hygiene and Environmental Medicine, Charité – University Medicine Berlin, Hindenburgdamm 27, 12203 Berlin, Germany; 2 German National Reference Centre for Surveillance of Nosocomial Infections (NRZ), Hindenburgdamm 27, 12203 Berlin, Germany

**Keywords:** Vancomycin-resistant enterococci, Multiresistant, Surveillance, Epidemiology

## Abstract

**Background:**

Enterococci are frequent pathogens causing nosocomial infections in Germany. Infections due to strains with vancomycin resistance are high when compared with other European states. Therefore, the study aimed to describe the recent progression of nosocomial infections due to vancomycin-resistant enterococci (VRE) in Germany.

**Methods:**

We analyzed data from two components of the German national nosocomial infection surveillance system for the period 2007–2016. For primary bloodstream infections (BSIs) and urinary tract infections (UTIs) we used data from intensive care units and for surgical site infections (SSIs) data from surgical departments. In a sensitivity analysis, we considered only data from participants that participated continuously from 2007 to 2016 (“core group”). We calculated proportions of VRE among all nosocomial enterococcal infections with 95% confidence intervals (95% CIs) and trends over time. A multivariable logistic regression was used to compare occurrence of VRE proportions among German federal states.

**Results:**

Enterococcal infections from 857 ICUs and 1119 surgical departments were analyzed. On ICUs, the proportion of vancomycin resistance in enterococci causing nosocomial infections significantly increased for BSIs from 5.9 to 16.7% and for UTIs from 2.9 to 9.9%; for surgical site infections, the proportion of VRE increased from 0.9 to 5.2% (*P* < 0.001 for all). In the core group, the increase of VRE was more pronounced in ICUs (BSIs: 5.5 to 21.6%; UTIs: 2 to 11.2%) but was not seen in surgical departments (SSIs: 1.5 to 2.8%). Compared with the most populous German federal state North Rhine Westphalia, enterococcal infections in Hesse (Odds Ratio (OR) 2.3, 95% CI 1.7–3.1), Saxony (OR 2.5, 95% CI 1.8–3.5) and Thuringia (OR 1.9, 95% CI 1.4–2.6) were more likely to be caused by vancomycin-resistant strains.

**Conclusion:**

In Germany, the proportion of VRE in nosocomial infection due to enterococci is still increasing. It remains unclear, why a large variation in the proportion of VRE exists between German federal states.

## Background

Vancomycin-resistant enterococci (VRE) have emerged as important multiresistant pathogens causing nosocomial infections [1, 2]. Infections with VRE are associated with increased length of stay and excess in-hospital mortality and therefore pose a rising public health threat [3]. In 2017, the World Health Organization identified VRE as one of the most important resistant bacteria in their “Global Priority list of antibiotic-resistant bacteria list” [4].

According to the 2016 surveillance report of the European Antimicrobial Resistance Network (EARS-Net), a significant increasing trend for VRE between 2013 and 2016 was not identified among invasive isolates for the EU/EEA population-weighted mean percentage [5]; however, in seven out of 30 participating countries a significant increase of VRE was observed. Ireland (VRE rate of 44.1%), Greece (27.9%), Slovakia (26.4%), Poland (25.2%) and Hungary (22.5%) showed the highest resistance rates in the EARS-Net 2016 report, if only countries with more than 100 reported isolates were considered. In Germany, the proportion of VRE among invasive *Enterococcus (E.) faecium* isolates was 12.1% and comparable with the EU/EEA mean.

Previously, we observed a dramatic increase of the proportion of VRE among blood-stream infections and surgical site infections between 2007 and 2012 [6]. Therefore, the objective of this study was to investigate the recent development of different nosocomial infections caused by VRE in Germany by using data from the large German national nosocomial infection surveillance system.

## Methods

We analyzed data that were recorded into two surveillance components of the German national nosocomial infection surveillance system (Krankenhaus-Infektions-Surveillance-System, KISS) between 2007 and 2016. Detailed information on KISS and its above-mentioned components have been described elsewhere [7]. Briefly, data on nosocomial primary blood stream-infections (BSI) and nosocomial urinary tract-infections (UTIs) on intensive care units (ICUs) were recorded in the ICU component of KISS (ICU-KISS). Data on nosocomial surgical site infections (SSIs) on surgical departments were recorded in OP-KISS. Infections were documented according to definitions by the CDC [8]. In KISS, enterococcal infections are not documented on a species level. Therefore, the term VRE is defined as all infections due to enterococci resistant against vancomycin regardless of the underlying mechanism or species.

### Ethics and data protection

We analyzed aggregated and anonymous data that are collected by the participating hospitals in accordance with the German “Protection against Infection Act” §23. Therefore, ethical approval by an institutional board was not necessary.

### Statistical analysis

We pooled data recorded in ICU-KISS and OP-KISS and analyzed the proportion of VRE for each type of infection (BSI, UTI, SSI) by dividing the number of enterococcal infections resistant against vancomycin by the number of all enterococcal infections multiplied by 100. 95% confidence intervals (CIs) were calculated. Data were univariate tested for a yearly linear trend by using Cochrane-Armitage-test [6].

A multivariable logistic regression was used to compare VRE proportions among German federal states. In addition to the risk factor federal state, the following potential confounders were considered: year of the surgical procedure, gender and age group for the patient (0–50, 51–65, 66–70 and 71–120 years), type of hospital (university hospital, other hospital), season, type of ICU or type of surgical department, and hospital size (400 beds and ≥ 400 beds). Stepwise forward-backward selection was used to derive the final logistic regression model. Parameters were entered into the model at a significance level of *P*  ≤  0.05 and were removed at *P*  >  0.05. Odds Ratios (OR) with 95% CIs were calculated.

Since not all hospitals reported data for the entire study period, we conducted a sensitivity analysis in which only hospitals were included that had reported data continuously from 2007 to 2016 for at least 6 month per year (“core group”). *P*-values less than 0.05 were considered statistically significant.

All analyses were performed with R 3.4.3 [R Core Team (2013); R Foundation for statistical computing, Vienna, Austria] and SAS 9.4 (SAS Institute Inc., Cary, NC, USA).

## Results

**Fig. 1:**
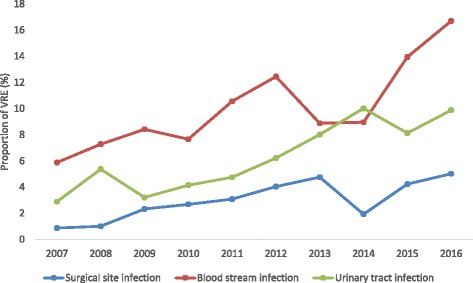
Time trend for percentage of vancomycin-resistant entercoccal (VRE) infections according to the German national nosocomial infection surveillance system (KISS), by infection site

Between 2007 and 2016 a total of 1121 ICUs and 1412 surgical departments from all German federal states reported data on nosocomial infections to ICU-KISS and OP-KISS, respectively (Table 1). Of those, 12,659 infections were due to enterococcus species. Overall, the proportion of VRE increased from 1.4% in 2007/2008 to 10% in 2015/2016 (Fig. 1). In BSI, the proportion of VRE increased from 5.9 to 16.7% (*P* < 0.001). Among UTIs and SSIs, the proportion of VRE increased from 2.9 to 9.9% (*P* < 0.001) and from 0.9 to 5% (*P* < 0.001), respectively (Table 2).

**Table 1:** ICUs and surgical departments providing VRE infection data for 2007–16 per year from the German national nosocomial infection surveillance system (KISS)

Year/number (*n*)	2007/08	2009/10	2011/12	2013/14	2015/16	Total^a^
Number (*n*) of ICUs	465	533	645	764	857	1121
Number (*n*) of surgical departments	432	558	681	919	1119	1412
Nosocomial enterococcal infections						
Total number (*N*) of nosocomial enterococcal infections	2047	2559	2253	2639	3161	12,659
Number of VRE infections, *n* (% (n/N))	79 (3.9)	106 (4.1)	143 (6.4)	187 (7.1)	318 (10.1)	833 (6.6)
Total number (*N*) of enterococcal infections on ICUs	1520	1927	1574	1700	1929	8650
Number of VRE infections on ICUs, *n* (% (n/N))	74 (4.9)	90 (4.7)	119 (7.6)	158 (9.3)	229 (11.9)	670 (7.8)
Total number (*N*) of nosocomial enterococcal infections in surgical wards	527	637	679	939	1232	4009
Number of nosocomial VRE infections in surgical wards, *n* (% (n/N))	5 (1.0)	16 (2.5)	24 (3.5)	30 (3.2)	57 (4.6)	132 (3.3)

*ICU* intensive care unit, *VRE* vancomycin-resistant enterococci; ^**a**^ Total number of ICUs/surgical departments that reported data for at least 6 month in any year

**Table 2:** Data on nosocomial infections due to Enterococci for 2007–16 per year from the German national nosocomial infection surveillance system (KISS), by infection site

	Surgical site infections			Bloodstream infections			Urinary tract infections		
Year	Enterococci (*n*)	VRE (*n*)	Proportion in % (95% CI)	Enterococci (*n*)	VRE (*n*)	Proportion in % (95% CI)	Enterococci (*n*)	VRE (*n*)	Proportion in % (95% CI)
2007	231	2	0.87 (0.15, 2.83)	170	10	5.88 (3.03, 10.23)	450	13	2.89 (1.62, 4.78)
2008	296	3	1.01 (0.26, 2.73)	206	15	7.28 (4.29, 11.47)	540	29	5.37 (3.69, 7.52)
2009	301	7	2.33 (1.02, 4.55)	273	23	8.42 (5.55, 12.18)	591	19	3.21 (2.00, 4.88
2010	336	9	2.68 (1.31, 4.86)	261	20	7.66 (4.88, 11.38)	627	26	4.15 (2.78, 5.93)
2011	357	11	3.08 (1.63, 5.29)	303	32	10.56 (7.46, 14.41)	379	18	4.75 (2.93, 7.26)
2012	322	13	4.04 (2.26, 6.63)	265	33	12.45 (8.88, 16.85)	434	27	6.22 (4.22, 8.80)
2013	420	20	4.76 (3.02, 7.13)	338	30	8.88 (6.18, 12.27)	462	37	8.01 (5.79, 10.76)
2014	519	10	1.93 (0.98, 3.41)	335	30	8.96 (6.24, 12.38)	439	44	10.02 (7.47, 13.10)
2015	615	26	4.23 (2.84, 6.05)	409	57	13.94 (10.83, 17.55)	504	41	8.13 (5.98, 10.77)
2016	617	31	5.02 (3.50, 6.97)	437	73	16.70 (12.39, 18.90)	496	49	9.88 (7.48, 12.75)

*VRE* vancomycin-resistant enterococci

**Fig. 2:**
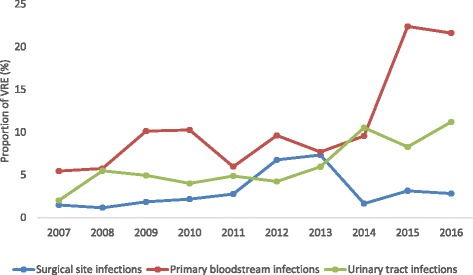
Time trend for percentage of vancomycin-resistant enterococcal (VRE) infections according to the German national nosocomial infection surveillance system (KISS), by infection site. Data from wards that participated continuously from 2007 to 2016 (“core group”)

According to our sensitivity analysis, 218 ICUs and 174 surgical wards reported data continuously on an annual basis (core group). Overall, the core group showed a comparable increase regarding the proportion of VRE from 4 to 10%; however, the increase in the core group was more pronounced in BSIs and UTIs whereas the proportion of VRE among SSI showed only a small increase (Fig. 2). VRE proportions increased significantly in primary BSI from 5.5 to 21.6% (*P* < 0.001) and in UTI from 2 to 11.2% (*P* = 0.001).

**Fig. 3:**
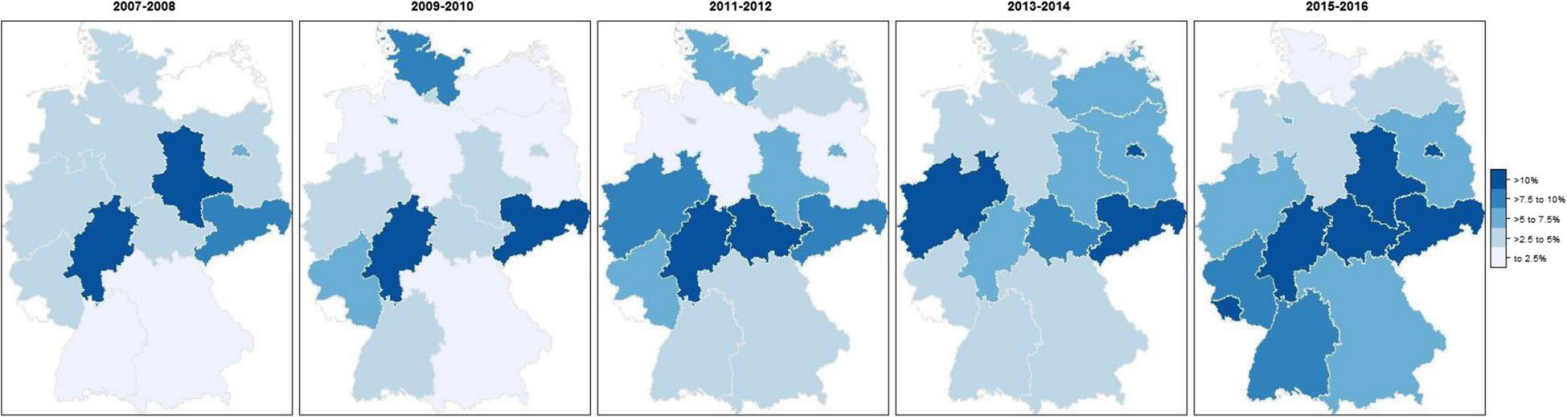
Distribution of the proportion of vancomycin-resistant entercoccal (VRE) infections among German federal states according to data from the German national nosocomial infection surveillance system (KISS) 2007–2016

Regarding differences in the proportion of VRE among German federal states, we found that proportion of VRE infections were > 10% in the 6 federal states of Berlin, Hesse, Saarland, Saxony-Anhalt, Saxony and Thuringia, all of which are in the center of Germany (Fig. 3). Compared with the most populous federal state North Rhine Westphalia, enterococcal infections in Hesse (OR 2.3, 95% CI 1.7–3.1), Saxony (OR 2.5, 95% CI 1.8–3.5) and Thuringia (OR 1.9, 95% CI 1.4–2.6) were more likely to be caused by VRE. Additionally, the final multivariable logistic regression indicated that type of hospital (university hospital vs. non-university hospital: OR 2.1, 95% CI 1.7–2.5), type of ICU (internal medicine ICU vs. non-internal medicine ICU: OR: 1.8 (95% CI 1.5–2.2) and calendar year (OR 1.1, 95% CI 1.1–1.2) statistically significant increased the chance for VRE.

## Discussion

Our analysis of the German national nosocomial infection surveillance system revealed a continuous increase of VRE proportions in nosocomial infections caused by enterococci in Germany between 2007 and 2016. This development has become even more apparent since our last report [6].

The results on VRE are concordant with other reports from Germany. Behnke et al. showed in two consecutive national point prevalence studies that vancomycin resistance in *E. faecium* causing nosocomial infections increased from 10.2% in 2011 to 23.1% in 2016 [9, 10]. Another study which analyzed data on antibiotic consumption and antimicrobial resistance in German ICUs found a continuous increase in resistance against vancomycin in *E. faecium* between 2001 and 2016 [11]. Finally, comparable results were found in the national antimicrobial resistance surveillance project [12], where detection of *E. faecium* derived from blood-cultures has increased by up to 50% in recent years and proportion of isolates resistant against vancomycin increased to 11.9% [13].

Interestingly, the recent report by the EARS-net described a high but stable situation regarding the proportion of VRE among *E. faecium* isolates of 12.1% in Germany, which is comparable with the European population weighted mean of 11.8% for 2016 [5]. These differences might be caused by the following reasons. (i) In EARS-Net, a static representative sample of hospitals from defined regions deliver rates of BSI from all wards based on laboratory results whereas in KISS a continuously growing sample of hospitals and individual wards report different types of infections (UTIs, SSIs and BSIs). However, even when considering only ICUs that continuously reported data on BSI we observed a continuous increase of VRE. (ii) As compared to EARS-net, we considered not only BSI but also surgical site infections and urinary tract infections. However, when only BSI were analysed in our study, the increase in the last 3 years has been particularly pronounced and differences to EARS-Net data remain unclear. (iii) Antimicrobial susceptibility testing (AST) in many microbiological laboratories Germany was performed according to Clinical & Laboratory Standards Institute (CLSI) while EARS-net uses EUCAST-standards only [14]. This potentially could influence vancomycin resistance rates especially in low and medium-level resistance due to differing recommended AST methods and clinical breakpoints [15].

Risk factors that have been described for VRE colonization or infections are long periods of hospitalization, hemodialysis, immunosuppression as well as close proximity to patients infected or colonized with VRE [16]. The fact that enterococci are able to survive on environmental surfaces for long periods of time [16, 17] highlights the importance of adherence to hand hygiene practice to prevent transmission. But despite improved adherence to hand-hygiene and successful reduction of nosocomial infections in Germany [18, 19], VRE continues to gain importance when compared to other multidrug resistant gram-positive organisms [11].

Increased antibiotic consumption has been advocated as another important risk factor that influence spread of VRE [16, 20, 21]. Many reports have established an indirect or direct link between appearance of VRE and the consumption of certain antimicrobial groups but the connection probably is more complex [21]. Exposure to substances with a broad gram-negative and anerobic microbiological spectrum, but no coverage against *E. faecium* are believed to facilitate the colonization of the GI-tract with VRE [22]. Particularly carbapenems [20], 3rd generation cephalosporins [23] but also penicillin’s with beta-lactamase inhibitors such as piperacillin/tazobactam [24] are likely to trigger this process. In Germany, cephalosporins are now the most commonly prescribed in German primary care [25] and might have triggered VRE-selection in the outpatient setting already. This would highlight the need of antibiotic stewardship not only in the inpatient but also in the outpatient setting [26, 27]. Interestingly, a recent meta-analysis has not found a direct influence of measures within in-hospital antimicrobial stewardship and the occurrence of VRE; however, the number of included studies was limited [28].

Although differences of VRE are obviously among European countries [5], only few studies have assessed regional differences of VRE within the same country. In a Canadian study, in which the molecular epidemiology of VRE from invasive samples from the National Nosocomial Infection Surveillance Program (CNISP) was analyzed found an increase of VRE in western and central Canada [29]. The authors assumed that the clonal spread of certain sequence types might have been in part responsible for these findings, although a reliable explanation was not possible. Kullar et al. evaluated regional variations on VRE across blood, urine and wound sources in United States Hospitals 2015 and found significant differences among US states [30]. A potential explanation for these results was not discussed.

The reasons for the regional differences in our study are also unclear and possible reasons for higher VRE rates in the center of Germany are difficult to explain and are likely to be multifactorial [6]. One possible explanation might be regional variations in antibiotic usage in the ambulatory and/or the inpatient setting; for example, a large population-based study found among German federal states differences in outpatient antibiotic prescription rates [25] of fluoroquinolones and cephalosporines, substances that might influence VRE selection process [1, 16, 22]. Other reasons for the regional differences that have been discussed are differences in the proportion of VRE in the environment (e.g. higher proportion of VRE in farm animals) or the spread of new or clonal VRE strain in certain areas [6]. However, Willems et al. found genetic differences in hospital-acquired VRE isolates and community-acquired or animal isolates which makes an association between the environment and increased VRE rates in German ICUs unlikely [31].

Some limitations have to be acknowledged: (i) Since fulfillment of case criteria depends on diagnostic sampling and documentation there is a risk for underdetection of cases. However, using proportions of VRE for all nosocomial infections caused by enterococci might have reduce confounding effects for changes in the bacteriological diagnostic over time, since only pathogen-derived infections were included. (ii) We did not record below a genus level of enterococci; therefore, we were unable to rule out an increase of Enterococcus spp. with intrinsic glycopeptide resistance. However, this is less likely since national and international sources have reported an increase vancomycin-resistant *E. faecium*, while infections due to vancomycin-resistant *E. faecalis* and Enterococcus spp. with intrinsic glycopeptid resistance e.g. *E. gallinarum* remain rare [2, 32]. (iii) Since we used surveillance definitions to determine infections, in some cases of UTIs the identified VRE might have been a contamination rather than the infection-causing pathogen. (iv) Finally, we had no information regarding strain characteristics, therefore local or regional outbreaks cannot be excluded.

## Conclusion

To proportion of vancomycin-resistant enterococcal infections has emerged as a relevant threat for patients and the healthcare system in Germany. Particularly the increasing rate of VRE in blood stream infections pose a serious threat for patients. Although specific recommendations and efforts regarding prevention of nosocomial infections, transmission of VRE and improvement in antimicrobial prescription are in place in Germany, the proportion of VRE continues to increase. To this end, implementation of effective strategies are necessary in order to reduce the spread of VRE.

## Abbreviations

95% CI: 95% confidence interval
BSI: Blood stream infection
ICU: Intensive care unit
OR: Odds ratio
SSI: Surgical site infection
UTI: Urinary tract infection;
VRE: Vancomycin-resistant enterococcus
